# A Review of Large Language Models in Medical Education, Clinical Decision Support, and Healthcare Administration

**DOI:** 10.3390/healthcare13060603

**Published:** 2025-03-10

**Authors:** Josip Vrdoljak, Zvonimir Boban, Marino Vilović, Marko Kumrić, Joško Božić

**Affiliations:** 1 Department for Pathophysiology, School of Medicine, University of Split, 21000 Split, Croatia; josip.vrdoljak@mefst.hr (J.V.); marino.vilovic@mefst.hr (M.V.); marko.kumric@mefst.hr (M.K.); 2 Department for Medical Physics, School of Medicine, University of Split, 21000 Split, Croatia; zvonimir.boban@mefst.hr

**Keywords:** large language models, clinical decision support, medical education, healthcare administration, artificial intelligence

## Abstract

**Background/Objectives**: Large language models (LLMs) have shown significant potential to transform various aspects of healthcare. This review aims to explore the current applications, challenges, and future prospects of LLMs in medical education, clinical decision support, and healthcare administration. **Methods**: A comprehensive literature review was conducted, examining the applications of LLMs across the three key domains. The analysis included their performance, challenges, and advancements, with a focus on techniques like retrieval-augmented generation (RAG). **Results:** In medical education, LLMs show promise as virtual patients, personalized tutors, and tools for generating study materials. Some models have outperformed junior trainees in specific medical knowledge assessments. Concerning clinical decision support, LLMs exhibit potential in diagnostic assistance, treatment recommendations, and medical knowledge retrieval, though performance varies across specialties and tasks. In healthcare administration, LLMs effectively automate tasks like clinical note summarization, data extraction, and report generation, potentially reducing administrative burdens on healthcare professionals. Despite their promise, challenges persist, including hallucination mitigation, addressing biases, and ensuring patient privacy and data security. **Conclusions:** LLMs have transformative potential in medicine but require careful integration into healthcare settings. Ethical considerations, regulatory challenges, and interdisciplinary collaboration between AI developers and healthcare professionals are essential. Future advancements in LLM performance and reliability through techniques such as RAG, fine-tuning, and reinforcement learning will be critical to ensuring patient safety and improving healthcare delivery.

## 1. Introduction

Artificial intelligence (AI) has exhibited rapid improvement in recent years, with substantial potential for application in all aspects of medicine and healthcare. The most recent leap in AI capabilities and applications was demonstrated with the release of large language models (LLMs), such as ChatGPT 3.5 and 4 from OpenAI [1].

LLMs originate from the transformer neural network architecture [2], and they are (pre)trained on large amounts of internet and textbook data with the goal of predicting the next word (token) in a sentence (sequence) [3]. Moreover, recent state-of-the-art foundation models are multimodal, i.e., other than text, they are also trained on images, videos, and even audio (e.g., GPT-4o) [4].

This type of pretraining via self-supervised learning leads to impressive performances at a wide array of downstream tasks and benchmarks [5]. For example, the most capable models like GPT-4 and Claude 3.5 achieve very high scores in the Massive Multi-task Language Understanding (MMLU) benchmark (86.4% and 86.8%, respectively) [6,7]. The MMLU benchmark was designed to test the model’s understanding and problem-solving capabilities across multiple topics and domains (from mathematics and computer science to law and medicine). For reference, an expert-level human (a particular subject) on average, achieves a score of 89.8% [7].

Numerous aspects of medicine and healthcare can benefit from the use of LLMs. From the automation of administrative tasks to improving and personalizing education, enabling decision support tools, and others. Moreover, models like GPT-4 have demonstrated impressive capabilities on rigorous assessments such as medical licensing examinations, suggesting a robust foundation for medical reasoning, which is an essential component that enables later usage in medical education and decision-support [8,9].

Given the amount of time doctors currently spend on drafting medical documentation, a significant proportion of this time could be saved by incorporating LLMs into the process. With a proper and detailed prompt (text input provided by the user), LLMs are great for drafting documents with proper structure and filling them with relevant patient data provided in the context [10]. By automating aspects of this process, LLMs could significantly alleviate the administrative burden faced by clinicians, potentially enhancing efficiency and reducing burnout.

LLMs are poised to play a transformative role in medical education and as decision support systems [11,12]. They could serve as an on-demand knowledge base for less experienced practitioners, offering guidance that aligns with the latest medical standards and guidelines, especially when enhanced with techniques like retrieval-augmented generation (RAG) [13,14]. Such tools integrate real-time, up-to-date medical information (like the Up-to-date and Statspearls databases) and treatment protocols directly into the LLM’s responses, enriching the model’s utility and accuracy [13,15].

However, the integration of LLMs into clinical practice is not without challenges. Studies have shown mixed results regarding their effectiveness as decision support tools. For instance, while some research highlights their proficiency in generating accurate diagnostic and treatment recommendations based on clinical casebooks, other studies, particularly in specialized fields like precision oncology, indicate that LLMs may not yet achieve the reliability and personalized insight provided by human experts [12,16].

**Figure 1:**
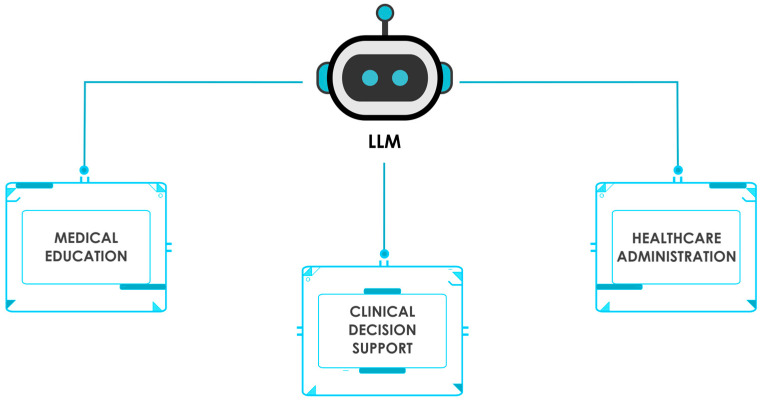
Schematic representation of LLM usage in key areas of healthcare.

In this comprehensive review, we will critically examine the current applications and future potential of large language models (LLMs) across three key healthcare domains: medical education, clinical decision support and knowledge retrieval, and healthcare administration (Figure 1). By synthesizing the latest research findings, we aim to provide a balanced assessment of the benefits, challenges, and limitations associated with LLM integration in these areas. Furthermore, we will discuss the ethical considerations and regulatory challenges that must be addressed to ensure the responsible deployment of LLMs in healthcare settings. Finally, we will explore emerging techniques and future directions for enhancing LLM performance, reliability, and safety, highlighting the importance of collaborative efforts between AI developers, healthcare professionals, and policymakers in realizing the transformative potential of LLMs in medicine while prioritizing patient well-being and the integrity of healthcare delivery.

## 2. Materials and Methods

The primary aim of this review was to examine how large language models (LLMs) are currently applied in medical education, clinical decision support (CDS) and knowledge retrieval, and healthcare administration. Specifically, we sought to (1) summarize the breadth of LLM-based tools and their reported efficacy, (2) highlight the most pressing challenges related to reliability, bias, and safety, and (3) discuss emerging techniques that might mitigate these limitations.

To find the relevant literature for this comprehensive review, two authors have independently conducted research in the following databases:

− PubMed (https://pubmed.ncbi.nlm.nih.gov/, accessed on 1 October 2024)
− ArXiv (https://arxiv.org/, accessed on 5 October 2024)
− IEEE Xplore (https://ieeexplore.ieee.org/Xplore/home.jsp, accessed on 2 February 2024)

By querying both a premier biomedical database (PubMed) and a preprint repository central to AI research (arXiv), the literature search balances clinical relevance with the newest technical advances in LLMs. arXiv includes leading-edge manuscripts on LLMs, often published months before they appear in a journal, giving a comprehensive look at both finalized and emerging LLM applications.

The following inclusion criteria were used:

- Articles discussing the use of LLMs in medical or healthcare settings
- Studies describing or evaluating LLM-based interventions or workflows in education, clinical decision-making, or administration
- Publications available in English
- Along with the following exclusion criteria:
- Papers lacking explicit mention of an LLM or focusing solely on AI methods not relevant to LLMs (e.g., non-transformer-based models)
- Abstracts without sufficient methodological detail
- Commentaries or opinion pieces that did not provide any empirical or explicit conceptual data

The following keywords were used in the search: (LLMs AND medicine) OR (LLMs AND healthcare) OR (LLMs AND medical decision support) OR (LLMs and medical education). The initial combined PubMed search yielded 613 studies, which we then filtered by performing a stepwise search. The search term (LLMs AND medicine) yielded 504 search results. The term (LLMs AND healthcare) yielded 216 search results, (LLMs AND medical decision support) yielded 55 results, and (LLMs and medical education) yielded 159 results. Searching for (LLMs and medicine) on arXiv yielded 117 results. On IEEE Xplore, the term (LLMs AND medical decision support) yielded 21 results, while LLMs and medical education yielded 53 results.

Relevant studies were filtered by title and abstract (and matched against our inclusion/exclusion criteria) to finally extract studies pertaining to LLM usage in medical education, clinical decision support, and healthcare administration. This approach has yielded a total of 22 studies we deemed the most relevant.

## 3. Large Language Models in Medical Education

LLMs have demonstrated significant potential to enhance multiple facets of medical education, including curriculum development, personalized study planning, and efficient literature review [17]. Studies collectively show that LLM integration can address common instructional challenges while reducing educators’ workloads, though limitations such as hallucinations, bias, and ethical concerns remain.

For instance, one single-site exploratory study found that embedding ChatGPT-3.5 within daily attending rounds improved knowledge gap coverage, facilitated initial diagnostic reasoning, and supported acute care decision-making [18].

Nevertheless, the authors underscore issues like misinformation, biases, and ethical implications. In a related review, Abd-Alrazaq et al. suggest that LLMs can serve as virtual tutors, generate case scenarios, and create personalized study plans but caution that “hallucinations”, or confidently stated inaccuracies, pose serious risks in a field where errors can have critical consequences [17,19].

Highlighting the depth of LLM knowledge, Bonilla et al. report that GPT-4-turbo outperformed lower-level radiation oncology trainees, reflecting its capacity to contribute to advanced learner instruction [20]. Still, other authors emphasize the potential for AI-driven overreliance and diminished critical thinking [21].

This concern aligns with findings by Arraujo and Correia, where students largely praised ChatGPT for generating ideas and rewriting text but voiced apprehensions about biases and responsible usage [22]. Furthermore, while ChatGPT excels at most knowledge-based assessments, it struggles with tasks requiring image interpretation and nuanced appraisal of the literature, illustrating domain-specific gaps [23]. This is highlighted by a study that evaluated ChatGPT’s performance on 50 different learning outcomes in Dental Medicine. ChatGPT accurately answered most knowledge-based assessments but struggled with image-based questions and critical literature appraisal, with word count being a notable limitation [23]. The struggle with the image-based questions is somewhat expected due to the relative lack of domain-specific image data (compared to text).

In addition to instructional support, LLM-enhanced platforms like MedEdMENTOR have shown moderate success in assisting researchers in selecting theoretical constructs, suggesting the potential to streamline scholarly endeavors [24]. The researchers have developed a custom GPT, which had access to an online platform for medical education (“MedEdMENTOR”), and investigated the usefulness of such a GPT system in helping medical researchers select theoretical constructs [24]. MedEdMENTOR AI was tested against 6 months of qualitative research from 24 core medical educational journals, where it was asked to recommend 5 theories for each study’s phenomenon. MedEdMENTOR AI correctly recommended the actual theoretical constructs for 55% (29 of 53) of the studies [24].

Finally, one potential use case for LLMs in medical education is in crafting multiple-choice question examinations for medical students, as was shown in a study by Klang et al. [25] (Table 1).

In this study, researchers have studied the medical accuracy of GPT-4 in generating multiple-choice medical questions. Out of 210 multiple-choice questions, only 1 generated question was deemed false, while 15% of questions necessitated revisions. While these results are promising, the study also highlighted important limitations. The AI-generated questions contained errors related to outdated terminology, age and gender insensitivities, and geographical inaccuracies, emphasizing the need for thorough review by specialist physicians before implementation. This underscores that while AI can be a valuable tool in medical education, human expertise still remains crucial for ensuring the quality and appropriateness of educational materials.

Similar findings were shown in another study that compared ChatGPT to humans in generating multiple-choice questions for the medical graduate exam [26] (Table 1). The researchers found no significant difference in question quality between questions drafted by A.I. and humans in the total assessment score as well as in other domains. However, the questions generated by A.I. yielded a wider range of scores, while those created by humans were consistent and within a narrower range. These studies highlight the potential of using LLMs to generate examination content. Pairing that with techniques like retrieval-augmented generation (RAG), where we also provide the relevant knowledge base to the LLM, could also improve the question relevance since the LLM would not rely only on its pretraining knowledge but also on the relevant domain data [27].

In summary, the studies reviewed in this section demonstrate the significant potential of LLMs in various aspects of medical education. From addressing knowledge gaps and supporting clinical decision-making to serving as virtual patients and personalized tutors, LLMs show promise in enhancing the learning experience for medical students and professionals. The integration of LLMs into medical education curricula appears to be well-received by students, offering benefits in content generation, exam preparation, and problem-solving. While LLMs like GPT-4 have shown impressive performance in certain medical domains, their limitations in areas such as image interpretation and critical analysis highlight the need for continued refinement and careful implementation. As the field progresses, further research is needed to optimize LLM use in medical education, addressing current limitations and developing best practices for their integration into existing educational frameworks. The development of specialized tools like MedEdMENTOR AI suggests a promising direction for tailoring LLMs to specific medical education needs, potentially revolutionizing how medical knowledge is acquired, applied, and evaluated in academic settings.

## 4. LLMs in Clinical Decision Support and Knowledge Retrieval

Large language models (LLMs) have demonstrated considerable promise in clinical decision-making and medical knowledge retrieval, as evidenced by multiple recent studies (Table 2). Collectively, these investigations highlight LLMs’ potential to assist clinicians in generating differential diagnoses, treatment plans, and evidence-based recommendations while also pinpointing persistent issues such as hallucinations, incomplete domain knowledge, and the need for robust oversight.

Several studies focus on augmenting LLMs with external knowledge repositories to enhance performance and reduce erroneous outputs. For instance, Wang et al. introduced a multi-step retrieval-augmented generation (RAG) pipeline that uses GPT-3.5 to rewrite user queries and filter relevant textbook passages before passing them to GPT-4 for final synthesis. This approach improved question-answering accuracy by up to 16.6% and significantly reduced hallucinations, underscoring the importance of structured domain knowledge in strengthening LLM outputs [28]. This study outlines the potential of using RAG for solving the common pitfalls of using LLMs in medicine (like the lack of domain knowledge and hallucinations).

Building on the theme of augmentation, Oniani et al. demonstrated that incorporating Clinical Practice Guidelines (CPGs) through methods such as Binary Decision Trees and Chain-of-Thought prompting improved both automated and expert evaluations of multiple LLMs, particularly in COVID-19 outpatient treatment [29]. These guideline-based methods illustrate how tailored frameworks can bolster reliability and factual consistency.

Other investigations use LLMs for specialized clinical tasks. Benary et al. evaluated four different models (ChatGPT, Galactica, Perplexity, and BioMedLM) for precision oncology decisions. Although the models generated a broad range of treatment options, their accuracy and recall trailed that of a human expert, reflecting limitations in complex or nuanced medical scenarios [12]. Moreover, LLMs produced at least one helpful option per case and identified two unique and useful treatments, suggesting the potential to complement established procedures. However, the study had limitations, including a small sample size and the use of fictional cases, which may affect the generalizability of the results [12] (Table 2).

The limitations of current open-source LLMs in clinical decision-making were further demonstrated in a subsequent study, which revealed significant performance gaps between these models and clinicians in patient diagnosis [30]. The research found that existing open-source LLMs (specifically Llama 2 Chat (70B), Open Assistant (70B), WizardLM (70B), Camel (70B) and Meditron (70B)) struggled to adhere to diagnostic and treatment guidelines and encountered difficulties with fundamental tasks such as laboratory result interpretation. The authors concluded that these models are not yet suitable for autonomous clinical decision-making and require substantial clinician oversight. However, it is important to note that both this study and the one by Benary et al. may not reflect the capabilities of the most recent open-source models, such as Llama 3 70b and 405b, which have demonstrated performance comparable to GPT-4 [30,31]. This rapid advancement in model capabilities highlights a persistent challenge in AI research: the potential for studies to become outdated during the publication process due to the accelerated pace of technological development. Consequently, the reported underperformance of open-source models may not accurately represent the current state of the field, as the latest iterations have shown marked improvements across relevant benchmarks.

Multiple proof-of-concept reports demonstrate moderate-to-high accuracy in specific disease contexts. ChatGPT, for example, aligned with NCCN Guidelines for head and neck cancer treatment in most simulated scenarios but still showed inaccuracies in more complex primary treatment cases [32]. Likewise, evaluating GPT-3.5 as a breast tumor board decision support tool revealed 70% agreement with an actual multidisciplinary team, although the authors caution that clinical oversight remains indispensable [32]. The study involved inputting clinical information from ten consecutive breast tumor board patients into ChatGPT and comparing its management recommendations to those of the actual tumor board. Results showed that ChatGPT’s recommendations aligned with the tumor board’s decisions in 70% of cases, with two senior radiologists independently grading ChatGPT’s performance favorably across summarization, recommendation, and explanation categories [33]. The researchers concluded that while these initial results demonstrate the potential for LLMs as decision support tools in breast tumor boards, clinicians should be aware of both the benefits and potential risks associated with this technology [33] (Table 2). Again, since GPT-3.5 was used, we can only expect that performance will be better with more recent models like GPT-4 and Claude-3.5. Further studies on a larger number of real-world examples should be conducted to obtain a more robust evaluation.

A parallel study in refractive surgery decision-making also showed a promising correlation between GPT-4 and a human expert, albeit with variability in responses and a need for larger-scale validation [34]. While the results were promising, the researchers noted limitations such as temporal instability, response variability, and dependency on a single human rater, emphasizing the need for further research to validate the use of LLMs in healthcare decision-making processes.

In another line of research, Stanford’s Almanac RAG-LLM system tackled frequent LLM issues, such as response variability and domain-specific knowledge gaps, by connecting to UpToDate and PubMed for real-time retrieval. Almanac outperformed standard LLMs on a newly created ClinicalQA benchmark, achieving stronger factuality, completeness, and user preference scores [35] (Table 2).

All aforementioned studies describe a common theme: how LLMs show promise as clinical decision support tools, given their accuracy in providing the correct diagnosis and treatment recommendations (Table 2). Furthermore, most of the studies also state the same limitations, such as LLM hallucinations, lack of domain-specific knowledge, and a lack of LLM response consistency. We already have solutions to these limitations, as can be seen in the works by Wang et al. and Zakka et al., where incorporating vector embeddings and semantic search to retrieve relevant medical knowledge (from up-to-date databases), along with other methods like knowledge graphs, reranking models, and query transforms [28,35]. With the ongoing release of advanced large language models (LLMs) incorporating novel algorithmic enhancements, it is reasonable to anticipate continued improvements in clinical performance. Consequently, the integration of LLMs into real-world clinical workflows can be expected in the foreseeable future.

**Table 2:** Large Language Models (LLMs) in Clinical Decision Support and Knowledge Retrieval.

Title	Authors/Year	Key Findings	Limitations
Augmenting Black-box LLMs with Medical Textbooks for Clinical Question Answering	[28]	Augmenting LLMs with comprehensive RAG pipelines leads to improved performance and reduced hallucinations in medical QA	The study only evaluated the system on three medical QA tasks without testing its performance on more complex clinical scenarios or real-world medical applications. Additionally, while the system showed improved accuracy, it relied on existing medical textbooks, which may become outdated, and the study did not assess the system’s ability to handle emerging medical knowledge or novel clinical cases.
Leveraging Large Language Models for Decision Support in Personalized Oncology	[12]	LLMs show potential in Personalized Oncology, albeit still not matching human expert level quality	The study was restricted to only 10 fictional cancer cases and used only 4 LLMs (ChatGPT, Galactica, Perplexity, and BioMedLM) for evaluation.
Evaluation and mitigation of the limitations of large language models in clinical decision-making	[30]	The researchers found that current state-of-the-art LLMs perform significantly worse than clinicians in diagnosing patients, fail to follow diagnostic and treatment guidelines, and struggle with basic tasks like interpreting laboratory results, concluding that LLMs are not yet ready for autonomous clinical decision-making and require extensive clinician supervision.	The study was restricted to only four common abdominal pathologies and used data from a single database (MIMIC) with a clear US-centric bias, as the data were gathered in an American hospital following American guidelines. Additionally, the study used only open-sourced Llama-2 based models.
Exploring the landscape of AI-assisted decision-making in head and neck cancer treatment: a comparative analysis of NCCN guidelines and ChatGPT responses	[32]	ChatGPT shows promise in providing treatment suggestions for Head and Neck cancer aligned with NCCN Guidelines	The study was restricted to hypothetical cases rather than real patient scenarios and only evaluated ChatGPT’s performance against NCCN Guidelines without considering other treatment guidelines or real-world clinical complexities.
Large language model (ChatGPT) as a support tool for breast tumor board	[33]	ChatGPT-3.5 provides good recommendations when evaluated as a decision support tool in breast cancer boards.	The study used a very small sample size of only 10 consecutive patients, relied on only two senior radiologists for evaluation, and was limited to using ChatGPT-3.5 accessed on a single day (9 February 2023).
Exploring the Potential of ChatGPT-4 in Predicting Refractive Surgery Categorizations: Comparative Study	[34]	ChatGPT-4 achieves significant agreement with clinicians in predicting refractive surgery categorizations	The study relied on a single human rater for comparison and used a small sample size of only 100 consecutive patients.
Almanac—Retrieval-Augmented Language Models for Clinical Medicine	[35]	Almanac, a RAG-LLM system, significantly outperforms standard LLMs in ClinicalQA, while also providing correct citations and handling adversarial prompts	The evaluation was limited to a panel of only 10 healthcare professionals (8 board-certified clinicians and 2 healthcare practitioners) and compared Almanac against only three standard LLMs (ChatGPT-4, Bing, and Bard). While the study used 314 clinical questions across nine medical specialties, it focused primarily on guidelines and treatment recommendations without evaluating other aspects of clinical decision-making.
Enhancing Large Language Models for Clinical Decision Support by Incorporating Clinical Practice Guidelines	[29]	Binary Decision Tree (BDT), Program-Aided Graph Construction (PAGC), and Chain-of-Thought–Few-Shot Prompting (CoT-FSP) improved performance in both automated and human evaluations.	Methods tested on a relatively small sample synthetic dataset of 39 patients.

## 5. LLMs in Healthcare Administration

Large language models have the potential to automate numerous tasks in healthcare administration that currently take up a lot of clinicians’ time, such as clinical note-taking, drafting patient or diagnostic reports, and patient data summarization. What is more, LLMs could assist in accurately coding medical procedures and diagnoses for billing purposes, potentially reducing errors and improving reimbursement processes.

In this section, we will review studies that investigated the LLM use case in healthcare administration.

Huang et al. have investigated ChatGPT-3.5’s potential for extracting structured data from clinical notes [36]. In particular, ChatGPT-3.5 demonstrated high accuracy in extracting pathological classifications from lung cancer and pediatric osteosarcoma pathology reports, outperforming traditional NLP methods and achieving accuracy rates of 89% to 100% across different datasets [36]. The study highlights the potential of LLMs in efficiently processing clinical notes for structured information extraction, which could significantly support healthcare research and clinical decision-making without requiring extensive task-specific human annotation and model training (Table 3).

In another study, Wei et al. explored ChatGPT’s capability in converting COVID-19 symptom narratives into structured symptom labels [36]. The study found that GPT-4 achieved high specificity (0.947–1.000) for all symptoms and high sensitivity for common symptoms (0.853–1.000), with moderate sensitivity for less common symptoms (0.200–1.000) using zero-shot prompting [37]. The research demonstrates ChatGPT’s efficacy as a valuable tool in medical research, particularly for efficiently extracting structured data from free-text responses, which could accelerate data compilation and synthesis in future disease outbreaks and improve the accuracy of symptom checkers (Table 3).

Moreover, when investigating the feasibility of LLMs in clinical text summarization, Van Veen et al. found that in most cases, summaries from the best-adapted LLMs were deemed either equivalent (45%) or superior (36%) to those produced by medical experts, as evaluated by 10 physicians on completeness, correctness, and conciseness [38]. This research suggests that integrating LLMs into clinical workflows could significantly reduce the documentation burden, allowing clinicians to allocate more time to patient care while also highlighting the need for careful consideration of potential errors and safety implications (Table 3).

In another similar study, Liu et al. investigated the potential of ChatGPT in medical dialog summarization, comparing it with fine-tuned pre-trained language models like BERTSUM and BART [39]. While BART achieved higher scores in automated metrics, such as ROUGE and BERTScore, ChatGPT’s summaries were favored more by human medical experts in manual evaluations, demonstrating better readability and overall quality [38]. The study highlights the promise of LLMs like GPT-3.5 in automated medical dialog summarization while also emphasizing the limitations of current automated evaluation metrics in assessing the outputs of these advanced models (Table 3).

Other authors have investigated LLM’s ability to transform inpatient discharge summaries to a patient-friendly language and format [40]. The study found that LLM-transformed discharge summaries were significantly more readable and understandable, with lower Flesch–Kincaid Grade Levels (6.2 vs. 11.0) and higher PEMAT understandability scores (81% vs. 13%) compared to the original summaries [40]. While the results demonstrate the potential of LLMs in improving patient comprehension of medical information, the study also highlighted the need for improvements in accuracy, completeness, and safety before implementation, emphasizing the importance of physician review to address potential safety concerns (Table 3).

**Table 3:** Large Language Models (LLMs) in Healthcare Administration.

Title	Authors/Year	Key Findings	Limitations
A critical assessment of using ChatGPT for extracting structured data from clinical notes	[36]	ChatGPT-3.5 demonstrated high accuracy in extracting pathological classifications from lung cancer and pediatric osteosarcoma pathology reports, outperforming traditional NLP methods and achieving accuracy rates of 89% to 100% across different datasets.	The study only evaluated ChatGPT’s performance on two specific types of pathology reports (lung cancer and pediatric osteosarcoma), which may not be representative of its effectiveness across other medical domains or different types of clinical notes.
Adapted large language models can outperform medical experts in clinical text summarization.	[38]	Summaries from the best-adapted LLMs were deemed either equivalent (45%) or superior (36%) to those produced by medical experts.	The study only focused on four specific types of clinical summarization tasks (radiology reports, patient questions, progress notes, and doctor–patient dialog), which may not encompass the full range of clinical documentation scenarios.
Extracting symptoms from free-text responses using ChatGPT among COVID-19 cases in Hong Kong	[37]	GPT-4 achieved high specificity (0.947–1.000) for all symptoms and high sensitivity for common symptoms (0.853–1.000), with moderate sensitivity for less common symptoms (0.200–1.000) using zero-shot prompting.	The performance evaluation was limited to common symptoms (>10% prevalence) and less common symptoms (2–10% prevalence), potentially missing rare but clinically significant symptoms.
Exploring the potential of ChatGPT in medical dialogue summarization: a study on consistency with human preferences	[39]	ChatGPT’s summaries were favored more by human medical experts in manual evaluations, demonstrating better readability and overall quality.	The research relied heavily on automated metrics (ROUGE and BERTScore), which could be inadequate for evaluating LLM-generated medical summaries.
Generative Artificial Intelligence to Transform Inpatient Discharge Summaries to Patient-Friendly Language and Format.	[40]	LLM-transformed discharge summaries were significantly more readable and understandable when compared to original summaries.	Small sample size of only 50 discharge summaries from a single institution (NYU Langone Health).

The aforementioned studies demonstrate the significant potential of current large language models (LLMs) in healthcare administration. These findings suggest that the integration of LLMs into daily clinical workflows could substantially alleviate the administrative burden on physicians and other healthcare professionals. By automating tasks such as clinical note summarization, patient data extraction, and report generation, LLMs show promise in streamlining administrative processes, potentially allowing healthcare workers to allocate more time to direct patient care.

## 6. Mitigating Current LLM Limitations in Healthcare

In this section, we will more closely explore the techniques that can be implemented to mitigate current LLM limitations in the healthcare setting (such as hallucinations and the lack of domain-specific medical knowledge). Specifically, we will focus on retrieval-augmented generation (RAG), where we will outline what contributes to a successful RAG system and what its main functionalities are.

Retrieval-augmented generation (RAG) is a technique that allows the addition of semantically relevant context to the input/prompt we provide to LLMs. The main constituents of RAG are: (1) relevant knowledge base, (2) embedding models, (3) vector database, (4) search via semantic similarity. The relevant knowledge base represents the additional data we want to provide to the LLM’s context, e.g., books and research papers (pdf files) or external medical knowledge databases (like StatsPearls or UpToDate). Embedding models are specialized machine learning models that transform text into numerical vector representations that capture semantic meaning and relationships. In the medical domain, these models can be specifically fine-tuned to understand complex medical terminology and concepts, with applications ranging from disease diagnosis to patient risk stratification. The choice of embedding model is particularly important in healthcare applications, as it directly affects the quality and relevance of retrieved medical information, with domain-specific models often performing better at capturing the nuances of medical language.

A vector database functions as a specialized storage system that maintains both textual segments and their corresponding vector representations, facilitating subsequent retrieval during semantic search operations. The semantic search mechanism operates through a multi-step process: initially, the user’s query undergoes vectorization through an embedding model, followed by the computation of vector similarity metrics (utilizing dot product or cosine similarity calculation) between the query vector and the stored vector representations. This process culminates in the identification and retrieval of the most semantically relevant text segments, which are then incorporated into the LLM’s contextual window. The mathematical formula for similarity computation can be expressed as:

cosine similarity=(a⃗·b⃗)/(|a⃗||b⃗|)|a⃗||b⃗|

Next, we will provide a specific example of how RAG might be used to improve LLM performance in the clinical decision-making setting. For example, as input, we have patient data for a particular visit (signs and symptoms, physical examination results, laboratory results, and other diagnostic procedures), and based on this input, we want the LLM to provide support in differential diagnosis and therapy recommendations. The RAG system can be connected to an external database like UpToDate and, based on the similarity of input text (e.g., signs and symptoms), fetch and provide the most relevant text chunks from the database, which can then provide the LLM with additional hints of what diagnosis might be considered given a specific set of symptoms. Then, in the next step, after the LLM provides the differential diagnosis, we can do another round of RAG and fetch the latest therapy guidelines for a given diagnosis, which are finally synthesized and provided to the user as the LLM’s response.

We also provide a code example of performing RAG for healthcare by connecting the LLM to an open-source StatsPearls database from PubMed (as inspired by Xiong et al.) [15]. We first preprocessed 2332 StasPearls articles as jsonl files, after which we embed them to a vector database utilizing open-source embedding model form “HuggingFace” (“all-MiniLM-L6-v2”), the “Langchain” library, and GPT-4 from OpenAI for response synthesis. Finally, we showcase how such a RAG system can be used for improving patient care in a primary physician’s office setting, where the initial input is given by ICD-code diagnosis and current treatment the patient takes. Given the initial input, LLM first generates a set of questions about how to improve patient care for a given case, which are then used to extract the relevant text chunks from the StatsPearls database. Initial patient data and relevant retrieved content are then provided to the LLM’s context for synthesizing the final recommendations. The code and relevant data are available at https://github.com/vrda23/Medical-RAG-showcase (accessed on 1 February 2025.).

## 7. Ethical Considerations and Regulatory Challenges

As with other high-impact technologies, LLM usage also raises major ethical concerns and regulatory challenges that need to appropriately be dealt with prior to successful integration into a real-world clinical practice. Firstly, we must take care of patient privacy and data security. This raises a concern in outsourcing patient data to close-source API providers like OpenAI or Anthropic, where patient data could be misused/used in future LLM training. Hence, hospitals could potentially host their own versions of open-source models and therefore not worry about patient data leakage. LLMs must be implemented in ways that ensure full HIPAA compliance and protection of sensitive patient information. Moreover, clear protocols are needed for data handling, storage, and transmission when LLMs are used to process patient records.

Also, questions remain about who bears legal responsibility when LLM-assisted decisions lead to adverse outcomes. For starters, the role of LLMs needs to be clearly defined as decision support tools rather than autonomous decision makers.

There is also the challenge of bias in medical pretraining data. LLMs can perpetuate or amplify existing healthcare disparities through biased outputs based on protected attributes like race, gender, or socioeconomic status. Studies show that even larger models or those fine-tuned on medical data are not necessarily less biased [41]. As a case example, Poulain et al. documented instances where LLM-based clinical decision support systems demonstrated lower accuracy in diagnoses for certain ethnic groups, highlighting how biased source data can translate into biased outputs [41].

Hence, proactive approaches to fairness in LLM development and deployment are needed to prevent exacerbating health inequities.

## 8. Conclusions and Future Directions

In contrast to current LLMs, there has been a new paradigm shift with the release of the latest OpenAI models, GPT-o1-preview and GPT-o1-mini [42]. While the exact mechanism is closed-source, these models implement reinforcement learning techniques that enable them effectively to “reason”. For each question, the models generate a long stream of reasoning steps (Chain-of-Thought), which enable longer test-time compute and, in essence, enable the model to spend more compute to “think” about a certain problem [42]. This has led to impressive performance in areas like math, coding, and formal logic. The models also show great promise in healthcare, especially in areas that benefit from improved reasoning capabilities, such as clinical decision support [43]. This new paradigm in model pre-training extracts the most benefit in already mentioned areas like math and coding where a clear reward signal can be derived. We argue how that can also hold true for medicine, where a clear reward signal (i.e., gold standard solution) can also be derived from, for example, diagnosis, prescribed medication, and exact dosage, based on specific patient cases. This could, in theory, lead to above-expert performance in certain subfields of medicine, or medicine in general, as similar underlying techniques were used in achieving superhuman performance in AlphaGo and chess [44].

By reviewing the current state of the evidence of LLM usage feasibility in medical education, clinical decision support, and knowledge retrieval, as well as in healthcare administration, we can conclude that LLMs hold great potential and will most likely be integrated in real-world workflows in the near future. Common pitfalls that postpone current LLM integration into clinical workflows, such as hallucinations and lack of specific knowledge, can be mitigated with already mentioned techniques like RAG via vector retrieval or knowledge graphs. Another possible venue to explore is LLM fine-tuning, which could also improve performance at specific downstream tasks (e.g., medical information extraction or specific patient report draft generation) [45]. Moreover, simply scaling the LLMs and using better-curated medical pretraining data should also lead to improved performance across all medical benchmarks.

In concluding remarks, LLMs present both significant opportunities and notable challenges in reshaping the future of medicine. Their potential to streamline administrative duties, enhance educational programs, and assist with clinical decision support is becoming increasingly evident. However, safe and effective implementation depends on thorough validation and robust oversight. With concerted efforts aimed at addressing ethical, technical, and regulatory hurdles, LLMs are poised to become invaluable partners in improving patient care and optimizing clinical workflows.

## Figures and Tables

**Table 1:** Large Language Models (LLMs) in Medical Education.

Title	Authors/Year	Key Findings	Limitations
ChatGPT as a Tool for Medical Education and Clinical Decision-Making on the Wards: Case Study	[18]	ChatGPT showed potential for addressing medical knowledge gaps and building differential diagnoses during ward rounds	Single-site exploratory evaluation with a small sample conducted over only 7 days at one urban academic medical center, using only ChatGPT-3.5 and relying primarily on qualitative phenomenological inquiry methods for analysis.
Large Language Models in Medical Education: Opportunities, Challenges, and Future Directions	[17]	LLMs offer a wide range of applications, virtual patient and tutor acting, generating medical cases and personalized study plans	A perspective/opinion paper that primarily draws on professional experience rather than empirical evidence, lacking systematic data collection or analysis to support its conclusions about LLMs in medical education.
Large language foundation models encode clinical radiation oncology domain knowledge: Performance on the American College of Radiology Standardized Examination	[20]	GPT-4-turbo performed best on clinical radiation oncology questions, outperforming some resident physicians	The study only evaluated performance on a single year’s (2021) ACR Radiation Oncology In-Training Exam, lacking longitudinal assessment across multiple exam versions. Additionally, the evaluation was limited to multiple-choice questions without assessing the models’ reasoning capabilities or ability to handle more complex clinical scenarios.
Harnessing the potential of large language models in medical education: promise and pitfalls	[21]	LLMs like OpenAI’s ChatGPT can transform education by enhancing student learning and faculty innovation, though challenges include academic misconduct, AI overreliance, reduced critical thinking, content accuracy concerns, and impacts on teaching staff.	A narrative review without a systematic methodology for literature selection and analysis, lacking empirical data to support its conclusions.
Incorporating ChatGPT in Medical Informatics Education: Mixed Methods Study on Student Perceptions and Experiential Integration Proposals	[22]	The study found that most students were satisfied with ChatGPT, citing benefits for content generation, brainstorming, and rewriting text, with proposals to integrate it into master’s courses for enhancing learning and assisting in various academic tasks.	A low number of questionnaire responses, which may affect the generalizability of the findings and the robustness of student perceptions regarding ChatGPT’s use in medical informatics education.
ChatGPT-A double-edged sword for healthcare education? Implications for assessments of dental students	[23]	The study evaluated ChatGPT’s accuracy on various healthcare education assessments, finding it provided accurate responses to most text-based questions but struggled with image-based questions and critical literature appraisal, highlighting the need for educators to adapt teaching and assessments to integrate AI while mitigating dishonest use.	Using only text-based questions without image processing capabilities, relying on the free version of ChatGPT with word count restrictions, and lacking. validation of the assessment items’ quality or difficulty level before testing them with ChatGPT.
MedEdMENTOR AI: Can artificial intelligence help medical education researchers select theoretical constructs?	[24]	MedEdMENTOR AI accurately recommended the actual theoretical constructs for 55% of qualitative studies from 24 core medical educational journals.	The study only evaluated MedEdMENTOR AI’s performance on theoretical construct recommendations for qualitative studies from a 6-month period in selected journals, with a relatively small sample size (53 studies), and only tested the system’s ability to match previously used theories rather than assessing the appropriateness or innovation of its recommendations.
Advantages and pitfalls in utilizing artificial intelligence for crafting medical examinations: a medical education pilot study with GPT-4	[25]	GPT-4 demonstrated the ability to rapidly generate a large number of multiple-choice questions for medical examinations with a low rate of outright errors (0.5%) but still required human expert review to address issues such as outdated terminology, demographic insensitivities, and methodological flaws in about 15% of the questions.	The study was limited to a single examination format (210 MCQs) based on an existing template, relied on only five specialist physicians for evaluation, and was conducted over a brief two-month period (March–April 2023). Additionally, the study lacked a comparison of time and resource efficiency between traditional question–writing methods and GPT-4-generated questions.
ChatGPT versus human in generating medical graduate exam multiple choice questions-A multinational prospective study (Hong Kong S.A.R., Singapore, Ireland, and the United Kingdom)	[26]	ChatGPT demonstrated the ability to generate multiple-choice questions for medical graduate examinations that were comparable in quality to those created by university professoriate staff, with only minor differences in relevance, while producing these questions in a fraction of the time required by human examiners.	The study used a relatively small sample size of only 50 MCQs per group, relied on just two human examiners for comparison, and was restricted to questions based on only two medical textbooks. Additionally, the wider range of scores in AI-generated questions suggests inconsistent quality, and the study did not assess the long-term reliability or validity of the AI-generated questions.
